# Advancing regulatory science through real-world data and real-world evidence

**DOI:** 10.1017/cts.2024.501

**Published:** 2024-05-21

**Authors:** Pablo Cure, Joshua P. Fessel, Christopher M. Hartshorn, Scott J. Steele

**Affiliations:** 1 National Center for Advancing Translational Sciences, National Institutes of Health, Bethesda, MD, USA; 2 Center for Leading Innovation and Collaboration (CLIC), Clinical and Translational Science Program National Coordinating Center, University of Rochester Medical Center, Rochester, NY, USA

## Background and overarching categories for thematic issue

This special-themed issue of the Journal of Clinical and Translational Science will focus on the use of diverse sources of Real-World Data (RWD) and Real-World Evidence (RWE) to Advance Translational Science. The goal is to highlight research, activities, and processes for the translation of RWD from a range of sources into RWE that can enhance the full translational science continuum, ultimately improving the development, approval, adoption, and use of safe and effective medical products.

The manuscripts in this thematic issue have been written for a broad target audience of all those engaged in clinical and translational science. Manuscripts addressing the spectrum of sources and applications of RWD/RWE in translational science were considered, with the following being of particular interest: Use of electronic health records, registries, claims data and other data sources to generate RWE; Opportunities and challenges for Digital Health Technologies to generate RWE; Evolution of novel methods to accelerate the generation and use of RWD/RWE; Policy considerations for the use of RWD/RWE; Use of RWD/RWE to improve clinical trials trial designs; Data access, use, sharing and fit-for-purpose considerations; Emerging use cases for RWD/RWE in translational science; Infrastructure to support the generation and; Use of RWD/RWE in translational science.

## Use of RWD to inform trial designs and generate RWE to evaluate safety and efficacy [1–12]

Currently, most of the patient-derived RWD used in biomedical research resides in Electronic Health Records (EHRs). Even though their development was not intended for research purposes, the large volume of information, data richness, continuum of care data collection, and ability to be linked and integrated with additional data from medical encounters such as claims data, imaging, and laboratory information can provide a more comprehensive view of an individual and their health journey. It is known that utilization of EHR data can be highly valuable in postmarket safety monitoring and surveillance. RWD leading to RWE has also been used as external control arms in interventional studies where randomization is impractical or unethical, and in different types of non-interventional studies. In this thematic issue, Selker *et al.* describe how through a more controlled environment utilizing standardized study protocols, the information obtained from a single patient could also help elucidate wanted and unwanted treatment effects that could be used as RWE and inform clinical decision-making at the individual patient level or even more broadly, if data from other similar cases is aggregated [8]. A series of manuscripts in this thematic issue also describe and apply a causal roadmap that can serve as a guide for studies that generate RWE [1, 2, 11]. In this framework, it is important to consider setting *a priori* ground rules for the utilization of the data including, fit-for-use, data availability, data quality, study design and analysis, quality of the results, assessment of causality, and grade of evidence to ultimately generate high-quality and relevant information to regulators and other stakeholders for clinical and/or regulatory decision-making.

## RWD/RWE to advance health outcomes and address health disparities [13–20]

A goal for RWD and the resulting RWE is to equally benefit patients and populations. Equity in distribution and access to life-saving therapies is also necessary to achieve a broader benefit of RWE. Through collecting RWD from multiple hospitals, medical practices, and health systems, we are now able to capture data from a much larger, widely distributed, and more representative and diverse population sample. The utilization of RWD has the potential to lower the bar for participation in study protocols, making it more convenient for individuals and researchers while increasing data representativeness. However, we must remain vigilant to avoid past mistakes when it comes to inclusion of diverse populations in research and focus on education, community engagement, and returning results to participants in a tailored manner. In this context, the manuscript by Hamer et al, provides an example of how to address barriers to access to care and therapeutics during a public health crisis through multi-level dissemination strategies utilizing RWD [13]. Utilization of multi-prong approaches (including RWD) in primary care settings as described by Krist *et al*. [19] and tailoring strategies to community needs can positively impact recruitment of diverse populations. Also, analysis of state-level RWD can help elucidate health disparities and risk factors associated with poor health outcomes such as maternal mortality with resulting evidence incorporated into improving awareness, education, and assisting with resource allocation and policy making [14].

## Opportunities for DHTs to generate RWD/RWE [15, 21–23]

Digital health technologies (DHTs) offer a host of opportunities to change how healthcare is delivered, tracked, and disseminated. DHTs are defined by FDA as integrated systems that use computing platforms, connectivity, software, and/or sensors for healthcare. DHTs are a broad category of data gathering and analysis tools that, in recent years, have paved the way for clinical research, consumer health, and clinical applications. They offer facile and often high-fidelity ways to collect real-world, observational data that is obtainable in real time. Albeit they will continue to be a target as RWD gathering tools moving forward, considerations in their implementation and their ability to deliver rigorous RWE, remain. For example, DHTs that have been leveraged to date for observational data collection are not as rigorously validated as traditional diagnostics; implementation issues remain in terms of those that cannot afford or do not live in regions with appropriate infrastructure; and the technology development pipeline far exceeds the evidence base to support their use. None of these considerations are intractable hurdles to the field *writ large* yet do involve additional “growing pains” in terms of their general use as RWD sources. Yet there are many examples to point to as to how the field is maturing. For example, in this issue, Bautista *et al* discuss the state of photoplethysmography, an optical technique that enables measurement of parameters associated with cardiorespiratory function [21, 23]. They highlight the unique nature of this tool and the methods (contact vs contactless) used currently both in terms of their positive and negative attributes for specific use cases (*e.g.,* validation of measurement) and populations (*e.g.,* neonates vs adults). These types of translational science questions continue to be asked for the plethora of available DHTs and, steadily, are being addressed.

## Policy considerations for RWD/RWE [24, 25]

Several policy considerations arise in the context of collecting, sharing, and utilizing RWD. While a range of policies and processes have been adopted to help ensure informed consent, data privacy, and the ethical use of individuals’ data, this remains a challenging issue given the evolving nature of the collection and utility of RWD, and potential changing and diverse perspectives on privacy. To provide additional insight on this topic, Hendricks-Sturrup and Lu explore perspectives related to privacy and willingness to share RWD [24]. While participants were generally supportive of sharing prescription history and other types of RWD, there were concerns about sharing other sources of RWD and the potential for use of RWD by third parties without specific consent. This highlights a broader issue of both ensuring needed privacy protections (through policy and technology approaches) and improving education and transparency regarding data collection, use, and privacy practices. Continued efforts in these areas will help ultimately realize the broader benefits of RWD.

## Infrastructure to support generating RWE [25–28]

The global pandemic has taught us that critical infrastructure already needs to be in place when an urgent need arises. The universe of RWD that can result in RWE will continue to expand, and the infrastructure to enable the conversion of various data types (some of which may not even exist yet) into research-ready data will need to be nimble and flexible. In this Thematic Issue, readers will find higher-level perspectives on RWD infrastructure through lens of the Clinical and Translational Science Awards program [26–28], as well as examples of what can be accomplished when powerful RWD infrastructure is brought together with innovative thinking and scientific inquiry.

## Summary

RWD can now be generated from multiple sources and at a much faster pace than traditional and more standardized clinical research information data collection sources (such as traditional case report forms), thus having the potential to, more accurately and in real-time, mimic the health journey of individuals and enrich our data universe in healthcare and research. However, utilization, integration, infrastructure, representativeness, privacy, security, and data sharing remain critical aspects for high quality, equitable, and secure RWD. The amalgamation of manuscripts herein provides a broad picture of sources, utilization, applicability, infrastructure, challenges, and opportunities to obtain meaningful RWD and generate the necessary RWE to ultimately improve the health journey for all.
